
JOURNAL INFORMATION
==============================
NLM Title Abbreviation: Wirtschaftsdienst
Journal ID: Wirtschaftsdienst
NLM Journal ID: 101086612

Wirtschaftsdienst (Hamburg, Germany : 1949)

ISSN: 0043-6275

ARTICLE INFORMATION
==============================
PMCID: PMC7678249
PMID: 33250540
DOI: 10.1007/s10273-020-2789-x
Article ID: 2789
Article version: 1
Subjects: Ökonomische Trends

Corona-Effekte im internationalen Vergleich

Gern Klaus-Jürgen klaus-juergen.gern@ifw-kiel.de
1
Hauber Philipp philipp.hauber@ifw-kiel.de
2
1 grid.462465.7 0000 0004 0493 2817 Forschungsgruppe Intern. Konjunktur, Institut für Weltwirtschaft, Kiellinie 66, 24105 Kiel, Deutschland
2 grid.462465.7 0000 0004 0493 2817 Prognosezentrum, Institut für Weltwirtschaft, Kiellinie 66, 24105 Kiel, Deutschland
Electronic publication date: 2020 Nov 20
Print publication date: 2020
Volume: 100
Issue: 11
First page: 899
Last page: 900
Copyright: © Der/die Autor(en) 2020
License: Open Access: Dieser Artikel wird unter der Creative Commons Namensnennung 4.0 International Lizenz (https://creativecommons.org/licenses/by/4.0/deed.de) veröffentlicht.
Open Access wird durch die ZBW - Leibniz-Informationszentrum Wirtschaft gefördert.
License URL: https://creativecommons.org/licenses/by/4.0/

issue-copyright-statement: © The Author(s) 2020

==============================

In den OECD-Ländern ist die Produktion in der Corona-Krise nahezu überall stark gefallen, im 2. Quartal um etwa 10 %, nachdem sie bereits im 1. Quartal um 2 % zurückgegangen war. Das Ausmaß des Einbruchs unterscheidet sich zwischen den Ländern aber deutlich (vgl. Abbildung 1). Besonders stark ging die gesamtwirtschaftliche Aktivität in Großbritannien und Spanien zurück, wo das Bruttoinlandsprodukt (BIP) um fast 23 % einbrach, sowie Italien und Frankreich. Vergleichsweise moderat war der Rückgang vor allem in einigen OECD-Ländern in Asien wie Südkorea und Taiwan, wo die Ausbreitung des Coronavirus vergleichsweise früh und zielgenau bekämpft wurde.

Kennzeichnend für die Corona-Krise ist, dass der private Konsum – eine üblicherweise im Konjunkturzyklus vergleichsweise stabile Komponente – in den meisten Ländern stärker als das BIP eingeschränkt wurde. Zumeist wurde ein Rückgang um mehr als 10 % verzeichnet, in Großbritannien und Spanien sogar um annähernd 25 %. Die Investitionen gingen zwar in den meisten Ländern ebenfalls deutlich zurück, in der Regel aber nicht stärker als der private Konsum. Sowohl Importe als auch Exporte gingen drastisch zurück, vor allem in den Ländern, die wie Spanien, Italien und Frankreich einen hohen Anteil der Wertschöpfung durch den internationalen Tourismus erzielen, da in großem Umfang Dienstleistungsexporte entfallen sind. Der Staatskonsum blieb in den meisten Ländern relativ stabil. In Großbritannien und Frankreich ist er allerdings massiv zurückgegangen, was wohl auch auf statistische Effekte zurückzuführen ist.

Hinsichtlich der Betroffenheit der Wirtschaftsbereiche unterscheidet sich die Corona-Krise deutlich von früheren Wirtschaftskrisen. Die Produktion im Verarbeitenden Gewerbe, die üblicherweise die konjunkturelle Dynamik prägt, schrumpfte in den meisten OECD-Ländern zwar auch in dieser Krise stark. Ungewöhnlich ist aber der Einbruch bei den personennahen Dienstleistungen, die sonst im Konjunkturzyklus eher stabilisierend wirken. Hierzu zählen Handel, Verkehr und Gastgewerbe sowie sonstige Dienstleistungen (einschließlich Kunst, Unterhaltung und Erholung) (vgl. Abbildung 2). Besonders groß sind die Unterschiede zwischen den Ländern bei der Entwicklung des Baugewer-

Abbildung 1

**BIP-Entwicklung in ausgewählten OECD-Ländern im 1. Halbjahr 2020**

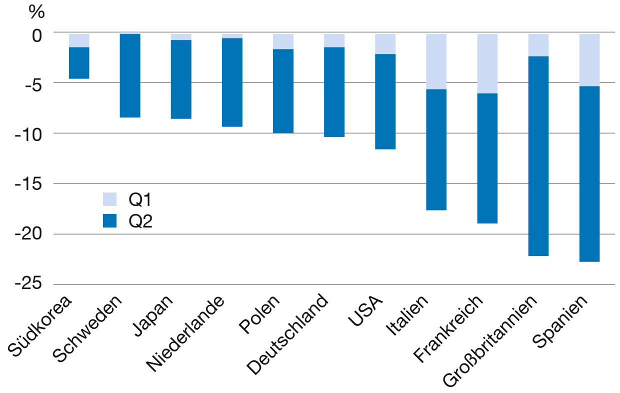

Anmerkungen: preis- und saisonbereinigt, Veränderung des Niveaus im 2. Quartal 2020 gegenüber dem 4. Quartal 2019 in %; Q1 und Q2: Beiträge der jeweiligen Quartale.

Quellen: nationale Statistikämter; OECD Main Economic Indicators.

Abbildung 2

**BIP-Entwicklung in OECD-Ländern im 1. Halbjahr 2020 nach Wirtschaftsbereichen**

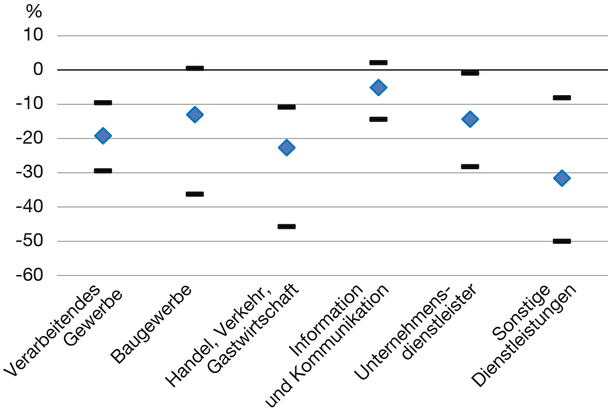

Anmerkungen: preis- und saisonbereinigt, prozentuale Veränderung des Niveaus im 2. Quartal 2020 gegenüber dem 4. Quartal 2019; die Raute zeigt den Durchschnitt, die schwarzen Balken Minimum und Maximum aus elf OECD-Ländern.

Quellen: nationale Statistikämter; OECD Main Economic Indicators.

Abbildung 3
**Zusammenhang zwischen wirtschaftlicher Aktivität und Lockdown-Strenge**

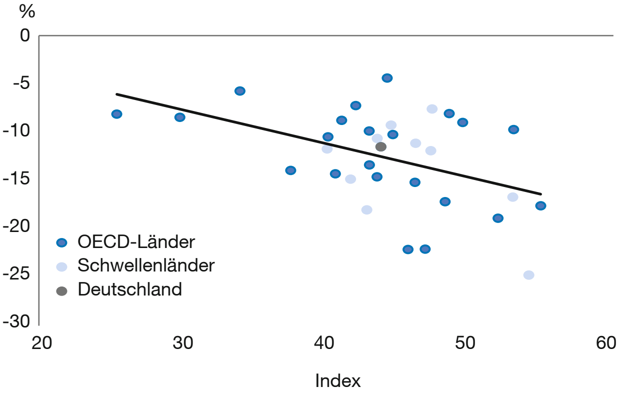

Anmerkungen: Daten für 33 Länder. y-Achse: Veränderung des BIP vom 1. Quartal 2019 bis 2. Quartal 2020. x-Achse: Lockdown-Strenge, Durchschnitt im 1. Halbjahr 2020, höhere Werte = strengere Maßnahmen.

Quellen: OECD Main Economic Indicators; Deutsche Bundesbank; Oxford Covid-19 Government Response Tracker.

bes. Beispielsweise kam die Bautätigkeit in einigen Ländern während des Shutdowns nahezu vollständig zum Erliegen (etwa in Frankreich und Spanien), in anderen Ländern (so in Deutschland) lief sie aber kaum gebremst weiter.

Zur Erklärung der Unterschiede in der Produktionsentwicklung zwischen den Ländern bieten sich verschiedene Faktoren an. Hierzu zählen das Infektionsgeschehen, die staatliche Einschränkung der wirtschaftlichen Aktivität und strukturelle Faktoren wie der wirtschaftliche Offenheitsgrad oder die Bedeutung der durch die Pandemie besonders betroffenen Wirtschaftsbereiche. Im Folgenden werden für 33 Länder, darunter sowohl fortgeschrittene Volkswirtschaften als auch Schwellenländer, Korrelationen des Rückgangs des BIP mit Indikatoren für die genannten Faktoren betrachtet (vgl. Tabelle 1). Zwischen der Infektionsrate und dem Rückgang des BIP zeigt sich kein signifikanter Zusammenhang. Eine stärkere Korrelation findet sich für die Zahl der Todesfälle pro Einwohner. Die Regierungen haben auf die Ausbreitung des Coronavirus mit zahlreichen Maßnahmen reagiert, welche die soziale Interaktion vermindern und damit die Infektionswelle brechen sollten. Das Ausmaß der Regulierungen unterscheidet sich von Land zu Land bezüglich des Zeitpunkts der Einführung, der Strenge der Maßnahmen sowie der Dauer ihrer Wirksamkeit. Ein statistischer Zusammenhang zwischen der staatlichen Reaktion und der Tiefe des wirtschaftlichen Einbruchs lässt sich mit Hilfe eines an der Universität Oxford entwickelten Index der Strenge der Maßnahmen (Stringency Index) zeigen (vgl. Abbildung 3). Ein struktureller Faktor, der die konjunkturellen Effekte beeinflussen könnte, ist die relative Bedeutung des Außenhandels für die Wertschöpfung. Denn Exporte und

Tabelle 1
**Zusammenhang zwischen ausgewählten Indikatoren und der wirtschaftlichen Aktivität im 1. Halbjahr**

| Indikator (unabhängige Variable) | Korrelationskoeffizient |
|---|---|
| Covid-19-Infektionen | -0,01 |
| Covid-19-Todesfälle | -0,37 (**) |
| Shutdown-Strenge | -0,45 (***) |
| Offenheit | 0,11 |
| Personennahe Dienstleistungen | -0,44 (***) |
| Verarbeitendes Gewerbe | 0,29 (*) |
| Tourismus | 0,49 (***) |

Anmerkungen: Daten für 33 Länder. Korrelation mit der Veränderung des BIP vom 1. Quartal 2019 bis 2. Quartal 2020. *, **, ***: Signifikanzniveau von 1 %, 5 % bzw. 10 %. Infektionen und Todesfälle: kumuliert, je 1 Mio. Einwohner*innen, Stand 30.6.2020; Shutdown-Strenge: Index, Durchschnitt im 1. Halbjahr 2020, höhere Werte = strengere Maßnahmen; Offenheit: Summe der Exporte und Importe im Verhältnis zur Wirtschaftsleistung; Personennahe Dienstleistungen: Wertschöpfungsanteile der Bereiche Transport, Handel, Gastgewerbe sowie sonstige Dienstleistungen; Verarbeitendes Gewerbe: Anteil des Verarbeitenden Gewerbes an der Bruttowertschöpfung; Tourismus: Gesamtbeitrag zur Wertschöpfung nach Angaben des World Travel and Tourism Council.

Quellen: OECD Main Economic Indicators; Deutsche Bundesbank; ECDC; IWF International Financial Statistics; Oxford Covid-19 Government Response Tracker; World Travel and Tourism Council.

Importe schwanken im Konjunkturzyklus typischerweise stärker als die Binnenkomponenten, und der Welthandel ist auch in der gegenwärtigen Krise eingebrochen. Zwischen dem Offenheitsgrad eines Landes und der Veränderungsrate des BIP zeigt sich allerdings kein Zusammenhang. Dazu passt, dass auch der Wertschöpfungsanteil des Verarbeitenden Gewerbes keinen nennenswerten Beitrag zur Erklärung der international unterschiedlichen Stärke des Abschwungs leistet, sondern sogar leicht positiv mit der wirtschaftlichen Aktivität korreliert. Hingegen ist ein höherer Anteil von Handel, Verkehr und Gastgewerbe zuzüglich sonstiger Dienstleistungen (einschließlich Kunst, Unterhaltung und Erholung), deren Aktivität in besonders hohem Maße und besonders dauerhaft unter sozialer Distanzierung leidet, in signifikanter Weise mit einem stärkeren Rückgang des BIP verbunden. Einen ähnlich großen Erklärungsbeitrag liefert der Wertschöpfungsanteil des Tourismussektors, wie er vom World Travel & Tourism Council ausgewiesen wird.

Klaus-Jürgen Gern, Philipp Hauber
philipp.hauber@ifw-kiel.de

Dieser Artikel ist eine gekürzte und überarbeitete Fassung des Abschnitts „Fokus: Pandemiegeschehen und gesamtwirtschaftliche Entwicklung im internationalen Vergleich“ im Gutachten der Gemeinschaftsdiagnose vom Herbst 2020, der unter Mitarbeit von Christian Grimme (ifo München), Geraldine Dany-Knedlik (DIW Berlin), Axel Lindner (IWH Halle) und Klaus Weyerstrass (IHS Wien) entstanden ist.

